# Supplementary material for: Complexome profiling of the Chlamydomonas psb28 mutant reveals TEF5 as an early PSII assembly factor
Source: Plant Cell. 2025 Mar 17;37(6):koaf055. doi: 10.1093/plcell/koaf055 (PMC12164590; doi:10.1093/plcell/koaf055)
Supplement: koaf055_Supplementary_Data [file koaf055_supplementary_data.zip › psb28_interactive.zip!/plots/intensity/qualityPlots/peptides/PETC_Cre11.g467689.t1.1.html]

### Cre11.g467689.t1.1

Trivial name: PETC  
SeqCoverage [median]: 32.00  
Mass: 21.52 kDa  
  
 

| Sequence | MissedCleavage | Mass [Da] | wtIntensity | muIntensity | Score |
| --- | --- | --- | --- | --- | --- |
| AAAASSEVPDMNKR | 1 | 1445.69 | 22442.76 | 26524.47 | 132.97 |
| AGEWLK | 0 | 702.37 | 113749.40 | 111464.74 | 138.38 |
| AGEWLKTHLAGDR | 1 | 1452.75 | 0.00 | 4336.44 | 77.53 |
| DALGNDIK | 0 | 844.43 | 449932.12 | 904155.15 | 168.08 |
| DALGNDIKAGEWLK | 1 | 1528.79 | 9294.69 | 62856.30 | 97.27 |
| GDPTYLIVTADSTIEK | 0 | 1721.87 | 71168.23 | 225382.56 | 158.25 |
| SLSQGLK | 0 | 731.42 | 233920.16 | 481584.86 | 173.11 |
| SLSQGLKGDPTYLIVTADSTIEK | 1 | 2435.28 | 44234.09 | 59411.44 | 137.64 |
| TGLEPWWA | 0 | 958.45 | 12534.42 | 0.00 | 106.29 |
| THLAGDR | 0 | 768.39 | 3624.49 | 34034.59 | 113.50 |
|  |  |  | 960900.4 | 1909750.6 |  |
